# Parasitic Nematode Immunomodulatory Strategies: Recent Advances and Perspectives

**DOI:** 10.3390/pathogens5030058

**Published:** 2016-09-14

**Authors:** Dustin Cooper, Ioannis Eleftherianos

**Affiliations:** Insect Infection and Immunity Lab., Department of Biological Sciences, Institute of Biomedical Sciences, The George Washington University, Washington DC 20052, USA; dcoop_13@gwmail.gwu.edu

**Keywords:** nematode, parasitism, immunomodulation, immune evasion, host innate immunity, adaptive immunity

## Abstract

More than half of the described species of the phylum Nematoda are considered parasitic, making them one of the most successful groups of parasites. Nematodes are capable of inhabiting a wide variety of niches. A vast array of vertebrate animals, insects, and plants are all identified as potential hosts for nematode parasitization. To invade these hosts successfully, parasitic nematodes must be able to protect themselves from the efficiency and potency of the host immune system. Innate immunity comprises the first wave of the host immune response, and in vertebrate animals it leads to the induction of the adaptive immune response. Nematodes have evolved elegant strategies that allow them to evade, suppress, or modulate host immune responses in order to persist and spread in the host. Nematode immunomodulation involves the secretion of molecules that are capable of suppressing various aspects of the host immune response in order to promote nematode invasion. Immunomodulatory mechanisms can be identified in parasitic nematodes infecting insects, plants, and mammals and vary greatly in the specific tactics by which the parasites modify the host immune response. Nematode-derived immunomodulatory effects have also been shown to affect, negatively or positively, the outcome of some concurrent diseases suffered by the host. Understanding nematode immunomodulatory actions will potentially reveal novel targets that will in turn lead to the development of effective means for the control of destructive nematode parasites.

## 1. Introduction

Nematodes, or roundworms, are a group of parasites that infect a wide range of hosts. Plants, insects and vertebrate animals all serve as hosts to several parasitic nematode species. Successful parasitization of a host requires both the penetration and invasion of host tissues, as well as persistence within the host’s body. The continued success of nematodes as parasites is, in large part, due to a multifaceted approach to survival within the host [1,2,3]. During the infection process, nematodes face a powerful innate immune system that regulates a variety of immune effector molecules. Survival and replication of nematode parasites in the host is achieved by suppressing, evading, or modulating the activity of certain host immune signaling pathways, as well as the immune functions that these pathways control [4,5].

Nematode immunomodulation is a strategy that promotes the survival of nematode parasites in the host by altering the activation of the host immune response during infection. This tactic involves the synthesis and release of molecules as excreted-secreted products into the host tissue during the invasion process. Molecules involved in nematode immunomodulation vary greatly depending on their mode of action and the type of host [6,7,8]. They include proteinaceous molecules, such as serpins and proteases, and small RNAs that are capable of mimicking host miRNAs [9,10,11,12,13,14]. While the effects of nematode immunomodulatory molecules are studied with respect to their contribution to nematode survival and killing of the host, they are usually broad-spectrum, and can impact host responses to concurrent diseases [15]. For example, molecules resulting in anti-inflammatory responses have been implicated in low prevalence of inflammatory bowel disease in populations with high incidence of nematode infections [16]. Conversely, nematode parasitism concurrent to malarial infection may modulate host immune responses against the latter [17].

While immunomodulation mechanisms are widespread among nematode parasites, very rarely are the mechanisms used against vastly different hosts taken together to display the equally broad variation observed among them [18]. This review summarizes recent progress on the identification and characterization of mechanisms used by parasitic nematodes to modulate various aspects of the host innate immune response of insects, plants, and vertebrate animals. Such information is invaluable for devising novel approaches to provide efficient protection to harmful parasitic nematode of humans or to determine mechanisms for enhancing the parasitic activity of beneficial nematode species that are used for the biological control of noxious insect pests.

## 2. Nematode Immunomodulation in Insects

Entomopathogenic nematodes are a group of roundworms that are able to infect and kill insects. To aid in this, they have developed an obligate mutualistic association with certain bacterial symbionts. This relationship is highly specific and has been a useful model to study the evolutionary origins of mutualism [19,20]. Research on entomopathogenic nematodes has focused on two genera: *Steinernema*, which associates with *Xenorhabdus* bacteria, and *Heterorhabditis*, which associates with *Photorhabdus* bacteria. Entomopathogenic nematodes provide shelter and serve as a vector for the bacteria, carrying them from one host to another. In return, once the nematodes have invaded an insect host, the bacteria are regurgitated and released within the insect hemocoel. The bacteria then secrete a cocktail of toxins and virulence factors and replicate rapidly in various tissues of the insect, the carcass of which is finally consumed by the nematode parasites [21,22,23].

Infection by nematode-bacteria complexes does not, however, go unnoticed by the insect host. The insect innate immune system has in place a series of mechanisms that promote the detection of the mutualistic partners in order to restrain their dissemination in the host [3,24]. Humoral and cellular immune responses make up the majority of the insect innate immune response [25,26]. Humoral immune responses are responsible for activating genes necessary for synthesizing and secreting antimicrobial peptides from the fat body (equivalent to the mammalian liver) into the hemolymph (insect blood) [27,28,29]. Cellular immune responses are regulated by the function of hemocytes (insect blood cells) [30]. During infection, hemocytes proliferate and contribute to the formation of cell aggregates, phagocytosis, and encapsulation, among other defense mechanisms [31,32]. The melanization response, which is regulated by the enzyme phenoloxidase, involves the synthesis and deposition of melanin that is directed against the invading microbes [33,34].

**Figure 1:**
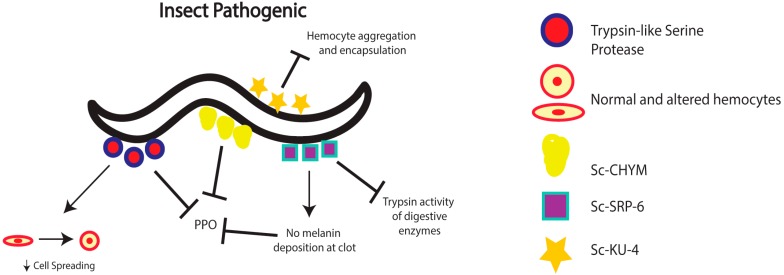
Insect Pathogenic nematodes modulate the phenoloxidase response via the secretion of a trypsin-like serine protease, Sc-CHYM, and Sc-SRP-6. The identified Trypsin-like serine protease has also been shown to limit spreading of insect hemocytes. Sc-SRP-6 modulates the host immune response by altering the activity of digestive enzymes. Sc-KU-4 acts by modulating the ability of hemocytes to aggregate or encapsulate foreign microbes.

During the course of infection within an insect host, nematodes must evade, suppress, or modulate the insect immune response in order to survive, expel their mutualistic bacteria, and complete several life-cycles [35]. Established mechanisms that nematodes employ to modulate the insect humoral and cellular immune reactions involve the production of molecules that are secreted by the parasites during the infection process. Proteases have been identified as a major contributor of host immunomodulation by entomopathogenic nematodes. These molecules can also aid in nematode invasion of the host and digestion of proteins within the infected insect [35]. Previous research has led to the purification and characterization of a trypsin-like serine protease that is secreted by *S. carpocapsae* infective juveniles during infection of *Galleria mellonella* larvae [36] (Figure 1). This protease was shown to possess a function in regulating immunomodulation and host immune inhibition, and thus protecting the nematode from the potent effects of the insect immune system. Following characterization, in vitro analysis of the trypsin-like serine protease revealed its prophenoloxidase inhibitory activity in a dose-dependent manner. In addition, incubation of hemocytes with the purified protease resulted in severe changes to the morphology of the cells. Untreated hemocytes were found to be flat, while treated hemocytes appeared round or spherical. The immunomodulatory activity also involved substantial decrease in the ability of hemocytes to perform cell spreading, a process whereby cells adhere to a foreign microbe and spread along the surface in order to phagocytose or encapsulate their target [37]. Further tests using *G. mellonella*-derived hemocytes co-incubated with three different entomopathogenic nematode species indicated that hemocytes were able to respond to *H. bacteriophora*, but not to *S. carpocapsae* or *S. glaseri*, suggesting that this kind of immunomodulation of insect hemocytes is probably specific to *Steinernema* nematodes.

*S. carpocapsae* nematodes also produce another molecule with immunomodulatory properties. The function of Sc-SRP-6, a novel serpin-like inhibitor, was investigated because of its elevated expression levels during the invasive stage of the nematode parasite [38], which suggested a potential role in nematode virulence (Figure 1). Functional analysis of Sc-SRP-6 revealed two roles in protecting the invading nematodes from the host immune response. First, Sc-SRP-6 inhibits the hydrolysis of food particles by reducing trypsin activity of the insect digestive enzymes. This finding indicates a role in the protection of *S. carpocapsae* from proteolytic enzyme activity. Second, Sc-SRP-6 was found to interfere with clot formation in infected insects [38,39]. In particular, binding assays revealed that Sc-SRP-6, together with three hemolymph plasma proteins, form complexes that prevent the incorporation of melanin into the clot matrix, an essential step in the encapsulation and nodulation immune processes [32,40]. Thus, this serpin-like inhibitor secreted by *S. carpocapsae* provides protection against insect digestive enzymes and participates in the inhibition of the melanization and encapsulation reactions against the nematodes.

Additional compounds with immunomodulatory characteristics have been identified and functionally characterized by examining the mixture of molecules produced by infective stage *S. carpocapsae* nematodes. One such compound, a chymotrypsin serine protease virulence factor designated “Sc-CHYM”, disrupts the activation of prophenoloxidase, which interrupts the melanization response and as a consequence prevents nematode encapsulation by hemocytes [41] (Figure 1). Another molecule, Sc-KU-4, has been identified as a Kunitz-family protease inhibitor [42] (Figure 1). The inhibitor was found to target insect immune recognition proteins in order to inhibit hemocyte aggregation, as well as the encapsulation of foreign particles. While these immune processes were functionally inhibited, it was also observed that Sc-KU-4 was not able to prevent their initial activation.

## 3. Nematode Immunomodulation in Plants

Plant-parasitic nematodes are capable of infecting and eliciting an innate immune response from their host plants [43,44]. Because of their drastic impact on food crops worldwide, a significant amount of research has gone into investigating the interactions between nematode strategies and molecules that are produced by the parasites during infection, and plant immune functions that are activated in response to nematode invasion [45]. Plant parasitic nematodes can be sedentary or migratory, with the latter causing extensive cellular damage to roots. It is the sedentary Heteroderidae family of nematodes, however, that are responsible for the majority of damage caused to food crops. The Heteroderidae family contains the cyst and root-knot nematodes, both of which employ similar strategies to parasitize plants. Motile, infective juveniles penetrate the root and induce permanent feeding sites. Once the nematodes begin feeding near the vascular tissue, they become sedentary and progress to the adult stage, during which they produce eggs [46]. The plant innate anti-nematode immune response relies on pattern recognition receptors to detect invading parasites in the extracellular matrix. Upon nematode detection, receptor-like kinases activate immune signaling pathways, giving rise to a variety of chemical defenses [47]. A common host defense is the programmed cell death of tissues surrounding the site of infection, preventing the spread of invading pathogens [48]. Modulation of host immunity by parasitic nematodes is necessary to prevent the programmed cell death of tissues surrounding the nematode feeding site, which would halt the growth and development of the parasite. The secretion of effectors capable of disrupting the receptor-like kinases or immune signaling pathways is an effective approach used by sedentary nematodes to aid in infection [49].

**Figure 2:**
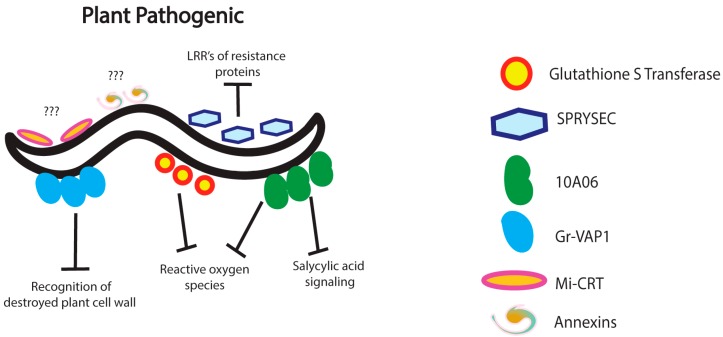
Plant Pathogenic nematodes use a multitude of strategies for host immunomodulation. The venom allergen-like proteins Gr-VAP1 can disrupt the host’s initial recognition of nematode invasion. Other proteins such as glutathione S-transferase or 10A06 interfere with reactive oxygen species produced in response to recognition of an invading nematode. 10A06 also interrupts salicylic acid signaling. SPRYSEC proteins interact resistance proteins to alter pathogen recognition. Some molecules, such as MiCRT, or groups of molecules, such as the annexins, are implicated in immunomodulation; however, their mechanism of action remains unknown.

*Globodera rostochiensis*, a potato cyst nematode, secretes a venom allergen-like protein, *Gr-VAP1*, concurrent with the secretion of enzymes that are responsible for the breakdown of the plant cell wall [50] (Figure 2). Because venom allergen-like proteins are conserved among a wide range of parasitic nematodes and are produced during the initial stages of parasitism, Gr-VAP1 was considered a potential modulator of the plant innate immune response. To investigate this, the effects of *Gr-VAP1* silencing on *G. rotochiensis* invasion and the heterologous expression of this protein in plants were observed. Knockdown of the venom allergen-like protein significantly impaired the ability of *G. rotochiensis* to successfully invade plant tissue, while expression of the protein in plants resulted in increased susceptibility of the plant to different types of pathogens. These results provided additional proof that Gr-VAP1 not only plays a role in, but also is required for nematode immunomodulation in plants. Further investigation indicated that Gr-VAP1 associates with proteases arising from the destruction of plant cell walls. These proteases are responsible for regulating signaling to pattern recognition receptors to activate immune signaling pathways, leading to programmed cell death of affected tissues. Through the secretion of Gr-VAP1, plant parasitic nematodes are capable of modulating this mechanism such that pattern recognition receptors are unable to recognize fragments of destroyed cell wall, thus preventing the induction of the plant innate immune response [50].

Modern advances in genomics and bioinformatics have allowed researchers to probe into the genomes of plant-parasitic nematodes in order to identify potential effectors produced and secreted into host plant tissues [51]. By investigating gene expression patterns in *G. rostochiensis*, researchers have discovered a novel gene family coding for proteins capable of interacting with plant resistance proteins [52]. One of the identified proteins secreted by these nematodes, called Secreted SPRY Domain-Containing Protein (SPRYSEC), was shown to interact with the leucine-rich repeat region of a coiled-coil nucleotide-binding pathogen resistance protein in tomato plants (Figure 2). It was hypothesized that this interaction promotes nematode virulence by modulating the activity of host immune receptor proteins. Transcriptome analysis of the root-knot nematode *Meloidogyne incognita* has revealed a group of genes that are up-regulated in the parasitic juveniles [53]. RNA-interference mediated silencing of the gene *Mi-gsts-1*, which encodes a glutathione S-transferase, revealed that the protein is produced in, and secreted by, the subventral oesophageal glands (Figure 2). The secreted enzyme is thought to modulate plant defenses in such a way that the nematode is protected from the negative effects of reactive oxygen species produced in response to parasitic attack. Similarly, the secreted effector protein 10A06 from the cyst-nematode *Heterodera schachtii* was found to protect the nematode from antioxidant plant-defenses during the infection process [54] (Figure 2). This protein is also thought to modulate salicylic acid defense signaling, thereby promoting nematode infectivity toward *Arabidopsis thaliana* plants. Although its specific mode of action has yet to be clarified, Mi-CRT, a calreticulin secreted by *M. incognita*, has been found to be important in nematode infectivity because silencing the expression of Mi-CRT reduces *M. incognita* virulence, whereas overexpression of this protein in *A. thaliana* increases sensitivity to the nematodes (Figure 2). It is thought that Mi-CRT is able to control the induction of plant defenses regulated by pathogen-associated molecular patterns [55]. More recent transcriptome analysis of infective juvenile *H. avenae* has shown the up-regulation of genes encoding annexins, a multigene family of well-known calcium-regulated proteins, capable of binding the phospholipids of cell membranes (Figure 2). Annexins are expressed in the oesophageal and sub-ventral secretory gland cells of plant parasitic nematodes and are potentially involved in nematode immunomodulation processes [56,57]. The same study also reports the identification of genes coding for fatty acid and retinol-binding proteins which may inhibit jasmonic acid synthesis and consequently restrict lipid-related plant defenses [56,58].

## 4. Nematode Immunomodulation in Vertebrates

Parasitic nematodes constitute one of the major threats to human health, causing diseases of major socioeconomic importance worldwide. Recent estimates indicate that more than one billion people are infected with parasitic nematodes, and more than a dozen species routinely parasitize humans [59,60]. The vertebrate innate immune response to parasitic nematodes is complex and varies greatly with the species and severity of nematode infection. Invasion of the host tissues by parasitic nematodes activates the complement system, a collection of proteins responsible for identifying pathogens and directing the innate immune response. Leukocytes are recruited to the site of infection and are responsible for enhancing the inflammatory response and release of cytokines, among a variety of other processes [61]. Mast cells and eosinophils participate in the innate immune response to nematode infection due to their role as potent effectors of a wide variety of cytokines and chemokines. Direct activation of leukocytes occurs via host tissue damage caused by the invading parasites [62]. Recruitment of other types of leukocytes, such as neutrophils, macrophages, basophils, innate lymphoid cells and dendritic cells, leads to the production of toxic free radicals, phagocytosis, and eventually, the development of an adaptive immune response [63,64]. However, similarly to insects and plants, certain vertebrate-parasitic nematodes have evolved immunomodulatory mechanisms that promote nematode infection by interrupting one or more effectors of the innate immune response [65].

**Figure 3:**
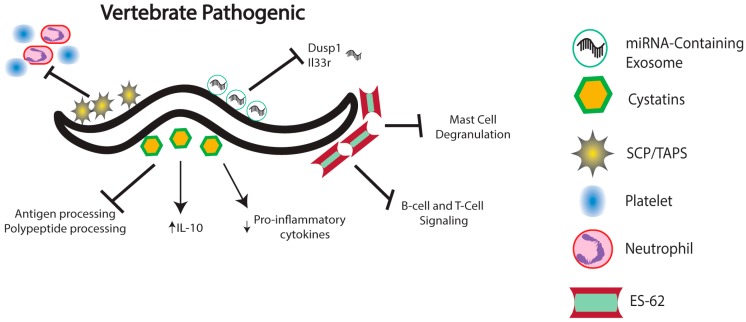
Vertebrate Pathogenic nematodes rely heavily on secreted cystatins for host immunomodulation. Cystatins influence the regulation of host cytokine production, as well as the development of adaptive immune responses by altering antigen and polypeptide processing. SCP/TAPS are believed to assist in inhibiting platelet and neutrophil activity. ES-62 alters a number of immune functions including B and T-Cell activation/proliferation via receptor signaling inhibition and the inhibition of mast cell degranulation through the formation of a complex with TLR-4. Recently, miRNA-containing exosomes secreted by some nematode species have been linked to altered host immune responses.

Cysteine protease inhibitors, or cystatins, produced by parasitic nematodes have been identified as a major class of molecules with immunomodulatory properties (Figure 3). Cystatins act in a multitude of ways to interfere with host effector mechanisms to prevent nematode clearance. Cystatins secreted by nematodes have been shown to inhibit two classes of cysteine proteases: legumains, which are responsible for antigen processing and presentation, and cathepsin L and S, which are involved in the processing of polypeptides. Inhibition of legumains is especially relevant to nematode infection because it prevents the generation of MHC class II molecules that recognize nematode antigens, thus preventing the induction of an adaptive immune response [66]. Nematode-secreted cystatins are also capable of modulating the host cytokine response in order to create favorable conditions for the invading parasites. More precisely, cystatins strongly enhance the production of the anti-inflammatory cytokine IL-10, which in turn restricts the activity of T cell-mediated responses [67]. A cystatin secreted by *Heligmosomoides polygyrus* modulates the activity of dendritic cells during the anti-nematode immune response in mice [68]. Interestingly, suppression of dendritic cell responses is a common feature of many mammalian parasites [69]. Dendritic cells exposed to recombinant cystatin expressed fewer MHC-II molecules as well as reduced CD40 and CD86, two proteins necessary for T-cell differentiation. The interference with antigen processing generated by the cystatin ultimately results in an immune response modulated in favor of nematode infection. Another cystatin secreted by *Acanthocheilonema vitae* modulates the proinflammatory effects of microglia by altering the expression of key cytokines [70]. Recombinant cystatin induces downregulation of iNOS as well as COX-2, both proinflammatory cytokines. It also induces the upregulation of IL-10, further promoting an anti-inflammatory effect upon microglia stimulated with lipopolysaccharide.

In addition, transcriptome profiling studies in mammalian parasitic nematodes have revealed molecules with known or predicted host immunomodulatory properties. The identified molecules include homologs of B-cell inhibitors, several serpins and neutrophil inhibitory factors in the barber’s pole worm *Haemonchus contortus* [71], as well as proteases that likely act by degrading host intestinal mucins and secretory leukocyte peptidase inhibitor related proteins with anti-inflammatory function in *Trichuris* whipworms [72]. Also revealed in these studies are several genes encoding a “sperm-coating protein-like extracellular domain” (also called SCP/Tpx-1/Ag5/PR-1/Sc7 or SCP/TAPS) in *Strongyloides* and *Trichostrongylus* nematode parasites [73,74] (Figure 3). SCP/TAPS have been proposed to participate in the inhibition of neutrophil and platelet activity as well as other immunomodulatory functions in hookworm infections [75,76].

The rodent filarial nematode *Acanthocheilonema viteae* secretes the glyprotein ES-62, which possesses immunomodulatory properties because it is able to interact with a variety of immune cells and is therefore capable of regulating the host immune system [77] (Figure 3). In particular, ES-62 interferes with molecular events that control B-cell and T-cell receptor signaling, which leads to the substantial inhibition of B cell and T cell activation and proliferation [78]. Also, ES-62 has an impact on antigen-presenting cells, such as macrophages and dendritic cells, and ES-62-mediated modulation requires the presence of TLR4, but not TLR2 and TLR6 [79]. In addition, ES-62 has been shown to inhibit mast cell degranulation by forming a complex with TLR-4 at the plasma membrane [80]. Impairment of the host immune response due to the multiple effects of ES-62 enhances nematode infectivity by promoting the survival and persistence of the parasites. Interestingly, infection by certain nematode species has been shown to slow down or circumvent the outbreak of various autoimmune or allergic related diseases, which is consistent with the theory of the ‘hygiene hypothesis’ [81]. A complete understanding of the molecular function of immunomodulatory molecules, such as ES-62, will potentially lead to the identification of novel anti-inflammatory drugs with distinct mechanisms of action [82].

There is also growing evidence for the involvement of exosomes in the regulation of several physiological processes including immunological function in response to various stresses [83] (Figure 3). Recent results indicate that certain parasitic nematodes synthesize exosomes that function by delivering small RNA molecules to mammalian cells to modulate the host immune response. It was recently shown that RNA secreted from *Heligmosomoides polygryus*, a nematode that infects the gastrointestinal tract of mice, contains 263 microRNAs (miRNAs), many of which are highly conserved throughout the nematode evolutionary history [14]. Interestingly, most of the identified miRNAs share seed sites with mouse miRNAs, suggesting that nematode-derived miRNAs may be capable of interacting with the mRNA targets of mouse-derived miRNAs. It was further determined that *H. polygyrus* miRNAs are secreted in vesicles also containing homologs for proteins of mammalian exosomes, and that these proteins can protect the vesicles from host degradation. Finally, in vivo analysis showed that nematode-secreted vesicles are internalized by mouse intestinal epithelial cells. This leads to changes in gene expression, most notably in the significant down-regulation of *Dusp1* and *Il33r*, both of which are implicated in mounting the type II innate immune response. Similar methods of immunomodulation have been identified for *Brugia malayi*, the nematode responsible for lymphatic filariasis [84], and species of *Onchocerca* nematodes, which parasitize cattle and humans [85]. Infective stage *B. malayi* release exosome-like vesicles containing miRNAs. These exosome-like vesicles are internalized by host macrophages, which leads to their activation [84]. By analyzing serum and plasma from humans and cattle infected with *O. volvulus* and *O. ochengi*, respectively, researchers were able to identify miRNAs that were potentially secreted by nematodes in a similar manner as described above [85]. These findings demonstrate the direct involvement of nematode-secreted exosomes containing miRNAs in altering several aspects of host immunity in order to promote the persistence of the parasites and the establishment of disease.

## 5. Conclusions and Future Directions

To be as prolific a parasite as nematodes are, they must evolve and employ robust mechanisms that promote their survival in the host. Immunomodulatory mechanisms are induced by parasitic nematodes to increase their infectivity once they have invaded their host. While by no means exhaustive, the mechanisms of nematode immunomodulation reported here display the variation of modulatory approaches developed by different types of parasitic nematodes to cripple the host immune response. While the general mechanism of nematode immunomodulation involves the production of molecules capable of altering certain host immune responses, the nature of the secreted compounds used by parasitic nematodes are drastically different. Despite great advances in our understanding of the mechanistic basis of nematode immunomodulatory processes, there exists a large number of parasitic nematodes that are able to infect different types of hosts, and that are capable of altering innate immune responses through the use of yet unidentified immunomodulatory molecules.

Identification and characterization of the molecular and functional basis of nematode immunomodulation will be an important step towards a better understanding of the range of mechanisms used by parasites to evade the host immune system in order to promote their survival, growth, and proliferation in the host. While elucidation of the mechanism(s) by which individual molecules operate is important, understanding how a specific nematode coordinates the activity and timing of different immunomodulatory processes will lead to an appreciation of the entire process of nematode parasitism. Determination of novel nematode immunomodulatory mechanisms may also provide unique and specific targets for developing novel anti-nematode therapies. For instance, eliminating crucial immunomodulatory molecules from human parasitic nematodes could allow the host immune response to function at full capacity, resulting in reduced nematode infectivity levels, and consequently rapid and efficient clearance of the parasites. Finally, a more complete understanding of the nematode immunomodulatory molecules that are involved in creating an anti-inflammatory environment in the host may contribute to the development of treatments for reducing the impact of inflammatory diseases [15].
